# Primary adenocarcinoma of the upper jejunum

**DOI:** 10.1002/jgf2.695

**Published:** 2024-07-05

**Authors:** Yuichi Sanada

**Affiliations:** ^1^ Department of Gastrointestinal Surgery Social Medical Corporation Taisei‐Kai Fukuoka Kinen Hospital Fukuoka Japan

## Abstract

Here we report a case of primary adenocarcinoma of the upper jejunum, for which an early diagnosis could be made by computed tomography.

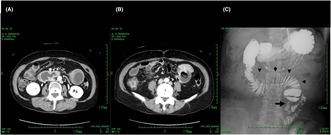

An 80‐year‐old woman with previous hypertension and hyperlipidemia presented to our department complaining of epigastric discomfort and nausea evolving over 7 days. She had no family history of malignant tumors or inflammatory bowel diseases. Her abdomen was slightly bloated and mildly tender in the epigastric region. She was diaphoretic with normal vital signs. Laboratory test results were notable for a leukocyte level of 10,200 cells/mm^3^ and an increased carbohydrate antigen 19–9 level of 51.8 U/mL.

Abdominal contrast‐enhanced computed tomography (CT) scan revealed a heterogenous circumferential mass at the upper jejunum (Figure 1A) with marked proximal bowel distension (Figure 1B). Esophagogastroduodenoscopy and total colonoscopy showed no malignant lesions. The upper jejunum could not be observed by endoscopy because of bile stasis.

**FIGURE 1:**
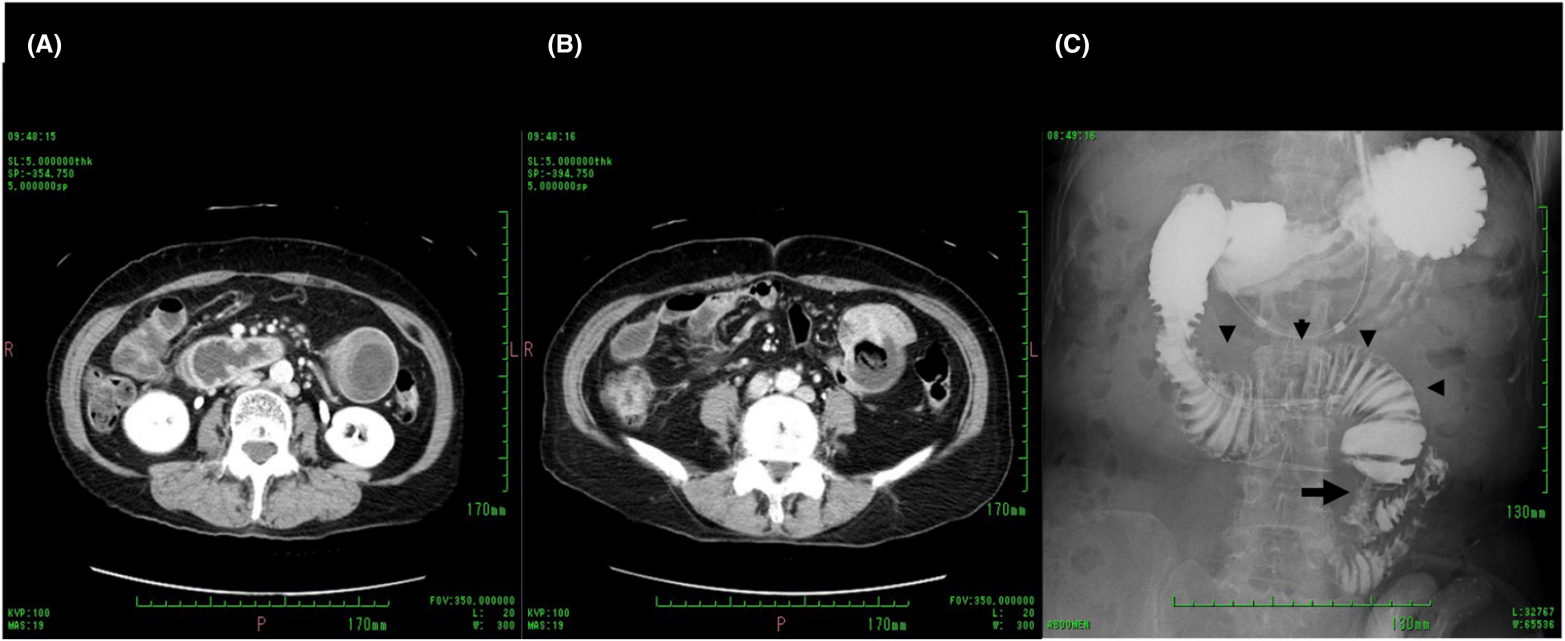
A contrast‐enhanced computed tomography image of the abdomen shows marked distension from the third portion of the duodenum to the jejunum around the ligament of Treitz (A). (A) circumferential tumor with heterogenous enhancement is shown at the upper jejunum (B). A contrast study through the nasojejunal tube shows localized stenosis of the upper jejunum (C, arrow) and dilation of the proximal intestine (C, arrowhead).

The gastrographin contrast study visualized an irregular stenosis of the upper jejunum with proximal bowel distension and stasis of the gastrographin (Figure 1C). The distal intestine was not distended. We therefore diagnosed a primary malignant tumor of the upper jejunum and treated her with decompression via nasojejunal tube and total parenteral nutrition for a week, followed by exploratory laparotomy.

The laparotomy revealed a 4 × 4‐cm round mass with a clear margin in the upper jejunum, 10 cm from the ligament of Treitz (Figure 2A). The mass involved the entire small intestine wall without direct invasion of the underlying mesentery. We performed partial jejunal resection with regional lymph node dissection limited to the neighboring mesentery, followed by end‐to‐end anastomosis. She had an uneventful postoperative course and was discharged 28 days after surgery.

**FIGURE 2:**
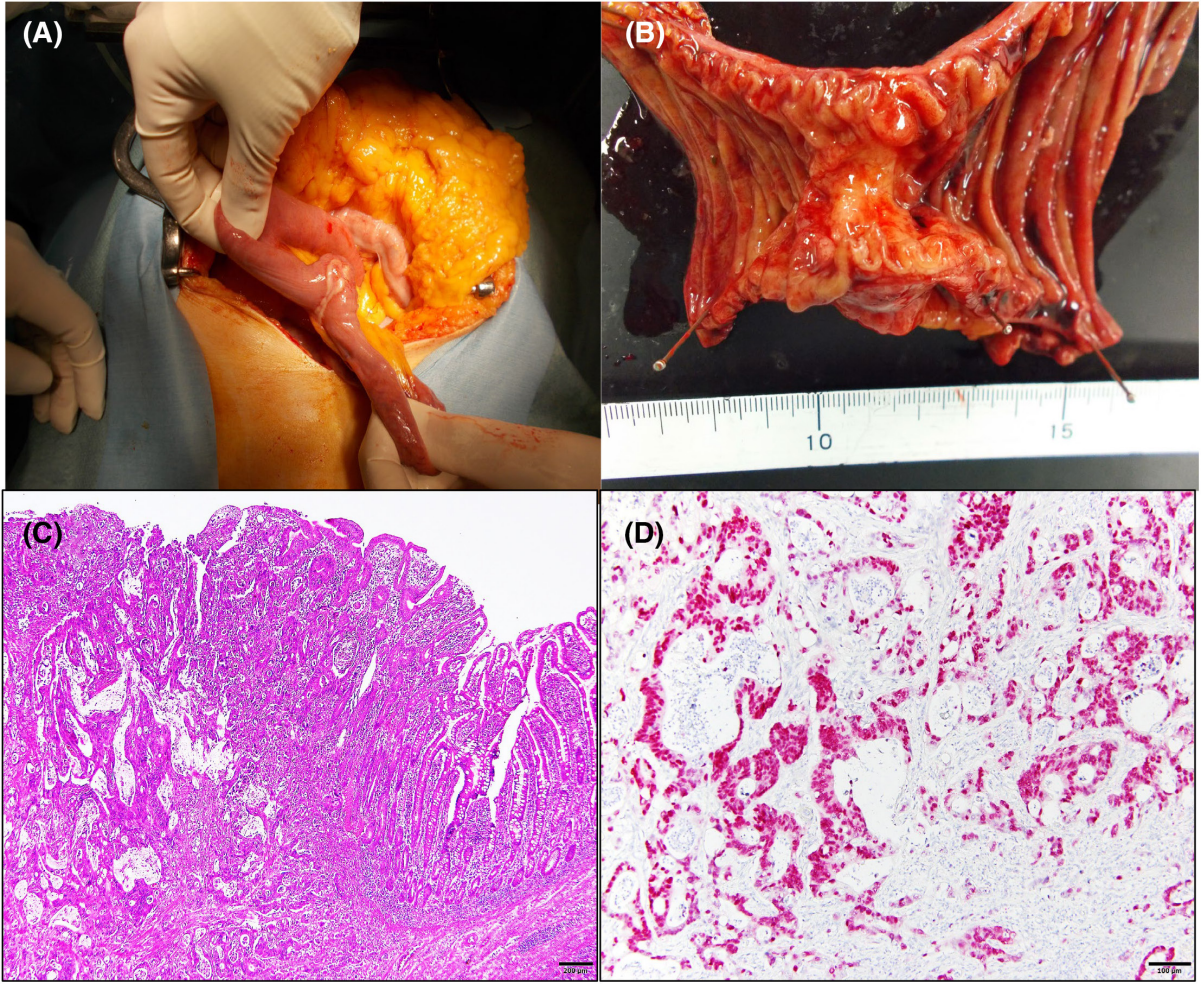
(A) At laparotomy, a 4 × 4‐cm round mass is identified at the upper jejunum, 10 cm from the ligament of Treitz. (B) The resected specimen shows an ulcerative tumor, 4 × 2.8 cm in size. (C) The histologic diagnosis is moderately differentiated adenocarcinoma with subserosal invasion. (D) Tumor cells diffusely express CDX‐2.

Grossly, the resected specimen appeared as an ulcerative tumor, 4 × 2.8 cm in size, with a clear boundary (Figure 2B). Histopathology showed moderately differentiated adenocarcinoma with subserosal invasion (Figure 2C). Most tumor cells diffusely expressed CDX‐2, suggesting an intestinal phenotype (Figure 2D).

Primary adenocarcinoma of the small intestine (SBA) is a rare tumor with a reported incidence of less than 5% among all gastrointestinal carcinomas.^1^ Nonspecific and site‐dependent symptoms of this disease can mimic other more common conditions, such as inflammatory bowel diseases, cholangitis, and colon carcinoma.^2^

Conservative treatments often incompletely relieve symptoms caused by SBA, jeopardizing early detection of this disease. Recent case reports have described patients with SBA receiving a diagnosis delayed by as much as 6–10 months, which affects prognosis.^3^, ^4^

Interestingly, our patient had been medically free of symptoms until 7 days before presentation, when epigastric discomfort and nausea developed.

Therefore, we considered this presentation as a case of “acute abdomen,” which prompted us to perform further imaging studies.

Contrast‐enhanced CT clearly visualized a circumferential tumor in the upper jejunum. We assume that acute, incomplete obstruction of the upper jejunum secondary to SBA caused epigastric discomfort that led to an early diagnosis. CT can detect SBA early, visualizing the primary lesion as a heterogeneously enhanced mass or distension of the proximal intestine.^5^

Awareness of SBA as a differential diagnosis for acute abdomen and imaging on initial presentation will yield an earlier diagnosis of this rare tumor.

## INFORMED CONSENT

Written informed consent was obtained from the patient for the publication of this clinical image.

## CONFLICT OF INTEREST STATEMENT

The authors have stated explicitly that there are no conflicts of interest in connection with this article.

## ETHICS STATEMENT

Ethical approval by the institutional review board of Fukuoka Kinen Hospital was not required for this report.
